# In transition with ADHD: the role of information, in facilitating or impeding young people’s transition into adult services

**DOI:** 10.1186/s12888-019-2284-3

**Published:** 2019-12-17

**Authors:** Anna Price, Tamsin Newlove-Delgado, Helen Eke, Moli Paul, Susan Young, Tamsin Ford, Astrid Janssens

**Affiliations:** 1 University of Exeter Medical School, St Luke’s Campus, Exeter, EX1 2LU UK; 2 Stratford CAMHS, Coventry and Warwickshire Partnership Trust, Stratford Healthcare, Stratford upon Avon, CV37 6NQ UK; 3 Honorary Associate Clinical Professor of Psychiatry, University of Warwick, Coventry, CV4 7AL UK; 4 Psychology Services Limited, London, UK; 5 Department of Public Health, University of Southern Denmark, J. B. Winsløws Vej 9B, DK-5000 Odense, Denmark

**Keywords:** ADHD, Transition, Information, Qualitative, Adolescent, Mental health

## Abstract

**Background:**

Many national and regional clinical guidelines emphasise the need for good communication of information to young people and their parent/carers about what to expect during transition into adult services. Recent research indicates only a minority of young people in need of transition for Attention Deficit Hyperactivity Disorder (ADHD) experience continuity of care into adulthood, with additional concerns about quality of transition. This qualitative study explored the role that information plays in experiences of transition from the perspectives of parent/carers and young people.

**Methods:**

Participants were recruited from 10 National Health Service Trusts, located across England, with varying service configurations. Ninety two qualitative interviews were conducted: 64 with young people with ADHD at different stages relative to transition, and 28 with parent/carers. Thematic analysis of data was completed using the Framework Method.

**Results:**

Interviewees reported a range of experiences; however reliance on parent/carers to gather and translate key information, and negative experiences associated with poor communication of information, were universal. Three themes emerged: *Navigating information with help from parents*; *Information on ADHD into adulthood*; *Information about the transition process.* The first revealed the essential role of parent in the translation and application of information, the other two explored distinct types of information necessary for a smooth transition. Interviewees made recommendations for clinical practice similar to UK (United Kingdom) National Institute for Health and Care Excellence (NICE) guidelines, with an additional emphasis on providing nuanced information on ADHD as a potentially long term condition. It was important to interviewees that General Practitioners had a basic understanding of adult ADHD and also had access to information about service provision.

**Conclusions:**

Our findings illustrate that the availability and communication of information to young people and their parent/carers is an essential component of the transition process between child and adult ADHD services. How and when it is provided may support or impede transition. This study constitutes a substantial contribution to the evidence base, drawing on interviews from a range of participants across England and from Trusts offering different types of services.

## Background

Attention Deficit Hyperactivity Disorder (ADHD), is increasingly recognised as a condition, which can persist into adulthood [1]. Treatment of ADHD in children can include parent-training, environmental modifications, medication, and cognitive behavioural therapy (CBT). For adults, treatments include medication and psychosocial / psychoeducational support to manage the impact of the condition [2, 3]. Mental health services in higher income countries are typically provided separately for children and adults [4, 5]. This means that young people with ongoing ADHD related healthcare needs are likely to need to transfer from child and adolescent mental health (CAMHS) or paediatric services into the care of different professionals, which may also be provided by a completely different organisation. Historically, ADHD was conceptualised as a disorder that only affected children, which was reflected in service provision [6–8], but as more studies demonstrated impairment into adulthood for some young people [9, 10], the need for continued health care services became evident. The 2008 National Institute for Health and Care Excellence (NICE) guidelines were the first in the United Kingdom (UK) to recommend treatment for adults [2].

UK NICE guidelines recommend that transition from child to adult services should take place as a supported process [11]. Transition in this context is defined as “the purposeful, planned movement of adolescents with chronic physical and mental conditions from child-centred to adult-orientated health care systems” [12]. Despite these recommendations, recent research indicates that only a minority of young people with ADHD who are in need of transition experience continuity of care into adulthood, with concerns about the quality of the transition [13–15].

The timing of transition from children’s services into adult mental health services (AMHS) occurs at a key developmental stage, when multiple other transitions such as changing educational setting, starting work for the first time or leaving home are likely to be taking place [4, 16]. Continued health care during this highly important life-stage can be pivotal in the outcome of these other important milestones, and is therefore crucially important [5]. Young people with ADHD may be particularly vulnerable to experiencing a disruptive transition due to a number of inter-related factors, which include the core symptoms of ADHD and the associated difficulties in organisation, variation in provision of adult ADHD services, negative and sceptical attitudes of some key professionals, and a lack of knowledge and training about ADHD in adulthood [17–22]. For young people who need ongoing support for their ADHD, a move to adult services may be experienced as disruptive and distressing, while the failure to complete transition into an adult service is likely to leave them without treatment. The resulting impaired functioning increases the associated adverse health, social, educational and occupational outcomes [5, 23–25], including increased risk of road traffic accidents [24], and higher rates of criminality [23].

The sharing of information with patients has long been recognised as a crucial component of health care; Coulter and Ellins [26] reported that information sharing has an impact on patients’ knowledge, understanding and experience of their condition, their use of services, and their general health behaviours. Active self-management of chronic conditions both for young people and adults often relies heavily on high quality information and communication practices [27, 28], yet concerns over the availability and accuracy of information are often raised in studies of ADHD [29, 30]. Misperceptions about the nature of the condition and its management are common both in wider society, and amongst young people with ADHD and their parents and carers [31–34]. ‘Misinformation’, such as believing that young people will definitely grow out of ADHD, or that medication is only needed to cope with school can have significant consequences. For example when examining adolescents’ decision making processes around medication, inaccurate information and beliefs were found to be related to non-adherence [35]. A lack of knowledge about ADHD as a condition that can persist into adulthood, and the management of adult ADHD, has also been reported amongst clinicians, which could translate into poorer provision of relevant and accurate information for young patients about what to expect from ‘living with ADHD’ [19, 32, 36].

Information and communication plays a prominent role in the recommendations from both the NICE [11] guidance on transition in general and the guidance on ADHD [3]. NICE recommends that young people and their parents and carers are given information about what to expect from adult services and what support is available to them; and that information about the young person is effectively communicated between child and adult services [3, 11]. The 2018 update to the NICE guidelines on management of ADHD includes a new section on ‘information and support’, which recommends that following diagnosis clinicians should conduct structured discussions that are tailored to meet individual needs and circumstances including relevance to stage of life, and on how ADHD may affect a patient’s life. However, the review of evidence informing the 2018 update [3], concluded that much of the supporting evidence was limited as most studies were conducted outside the UK, or were of insufficient depth or quality [37].

A recent systematic review of experiences of ADHD transitions synthesised existing qualitative evidence, with findings indicating that information played an important role, perhaps beyond that already identified in the NICE guidelines [30]. Included evidence highlighted the importance of communicating information about the young person between services and providing information on the transition process [30]. However previous studies have included small samples, covered a limited geographic area [38], included only healthcare professionals [22], or were consensus statements based on expert discussions [39]. To our knowledge this study is the first UK-based, in-depth exploration of the role of information in ADHD transitions from the perspective of young people and their parents at different stages relative to the age of transition, and from locations with varying service structures.

The aim of this paper is to explore from the perspectives of parent/carers and young people, the role that information plays in experiences of transition into adult ADHD services, how this impacts on transition outcomes and how it affects engagement and agency of the young person. It is based on an analysis of the qualitative data gathered during the ‘Young people with ADHD in transition from children’s services to adult services’ (CATCh-uS) study. CATCh-uS is a multi-strand research project on ADHD transitions, which included a qualitative study exploring young peoples’, their parent/carers’, and clinicians’ experiences of transition [40], from which information arose as an important theme.

## Methods

The National Institute of Health Research (NIHR) funded the CATCh-uS project which applied mixed methods to investigate transition from child to adult services for young people with ADHD [41]. CATCh-uS included a large qualitative exploration of stakeholders’ experiences of the transition process, which involved interviews with young people, parents, and clinicians. This study used a qualitative design, based on a portion of qualitative data from the larger study. The current paper presents a focused study on the role of information from these data. ‘Information’ was defined broadly to include processes for sharing and communicating information, types of information, and transferring of information, with emerging themes shaped by the data. Ethical approval for this element of the study was granted by NRES South Yorkshire Ethics Committee: Yorkshire & The Humber (REC Reference: 15/YH/0426) and the University of Exeter Medical School Research Ethics Committee (REC Application Number: 15/07/070). For full details of recruitment strategy please see the CATCh-uS report [40].

This paper focuses on two of these stakeholder groups: young people, and parents of young people with ADHD. To explore differences at each phase of the transition process, we recruited three groups of young people:

- in children’s services (*pre transition)*
- just transitioned directly from child to adult services (*at transition*)
- young adults diagnosed with ADHD in childhood who disengaged with services for at least a year before re-entering adult services (*no transition*)

A fourth group was comprised of parents of young people from each of the above groups.

### Sampling and recruitment

Both young people and parents were recruited via 10 participating National Health Service (NHS) provider organisations (Trusts). Five trusts were purposefully selected to capture regional variation as well as a range of service models for adults with ADHD, from specialist to generic AMHS (South London and Maudsley NHS Foundation Trust, Berkshire Healthcare Foundation NHS Trust, Devon Partnership NHS Trust, Coventry & Warwickshire Partnership Trust, Nottinghamshire Healthcare NHS Foundation Trust). Participants were also recruited from five other NHS Trusts that subsequently volunteered to recruit towards the study via the NIHR Clinical Research Network: these included South Staffordshire & Shropshire Foundation Trust, Leicestershire Partnership NHS Trust, Lincolnshire Partnership NHS Foundation Trust, Somerset Partnership NHS Foundation Trust, and Sussex Community NHS Foundation Trust.

We aimed to recruit 20 to 25 young people for each of the three groups of different ages and stages related to transition, as well as a similar number of parent/carers, henceforth referred to as parents. All parents had a child in services; dyads of parent and young person were accepted but not insisted upon. We recruited for all four groups using a sampling matrix to ensure variety in: location and type of service provision (with or without follow-up adult services for ADHD), gender, comorbidity, and residence of participant (with parents or elsewhere), and occupation (school; higher education; employment; or not in education, employment or training). We also aimed to recruit parents of children who were still with children’s services, had transitioned directly, or had experienced some time without services.

Recruitment of young people and parents was continuously monitored to ensure that the sampling frame was being evenly populated in line with sampling aims stated above, with a focus on harder to reach groups, such as young women. Eligible participants were approached by staff from their NHS Trust. Once participants had agreed for their details to be shared with the research team, they were contacted by a researcher to arrange an interview. Participants could choose how (face-to-face or via a telephone) and where they were interviewed (home, hospital, public place or over Skype) and whether or not a companion attended the interview.

Informed consent was gained from all participants aged 16 years and above. For participants under 16, their assent and the consent of a person with Parental Responsibility (as defined by the Children Act 1989) was gained. For all participants, written consent was documented prior to the interview and all young people were offered a £10 voucher as an incentive.

Decisions about sample size drew on our experiences of previous studies on transition and wider methodological findings regarding the anticipated stage in data collection when data saturation is likely to occur (Mitchell, 2014; Beresford, 2014).

### Interview procedure

AP, AJ and HE conducted semi-structured interviews using a topic guide (see Additional file 1) informed by existing literature on transition and the project’s parent advisory group and covered the following topics:

- current and future medication use,
- current and future contact with services,
- preparation for and/or experiences of the transition process,
- views on key elements of optimal transition.

All interviews were digitally voice-recorded and transcribed verbatim.

### Data management and analysis

Data analysis were conducted using inductive and deductive methods of thematic qualitative analysis, drawing on strategies proposed by Braun and Clarke [42]. We worked within a Framework method that has been specifically developed in the context of applied healthcare research [43]. Data collection and analysis of young people and parent interviews were split into two phases to allow for an interim analysis so that we could assess data saturation, refine the topic guide to reflect unanticipated emerging themes, and adjust the sampling frame to ensure that our final attained sample reflected all important stakeholder groups (phase 1: 1 April 2016 until 30 November 2016, Phase 2: 1 March 2017 until 31 May 2017).

Each recruited participant was assigned a unique identifier code; descriptive data on the participants were stored in an encrypted spreadsheet. Interview recordings and transcriptions were stored on an encrypted hard drive. Once transcribed, interview data were managed using QSR International’s NVivo12 qualitative data analysis software [44] and were stored securely and password protected. The interviews were analysed by the research team using thematic analysis with Framework [43]. This method facilitates systematic and transparent data analysis, and enables researchers to identify patterns or commonalities, as well as contradictions in and between participants’ accounts, so they can explore and test explanations for those patterns [45, 46].

The first stage of analysis involved ‘indexing’ a small sample of interviews, to gather an insight and overview of the data. A thematic framework or ‘coding tree’ was then created that identified key concepts and was used to code all remaining interviews. The next stage involved writing summaries of each interview for every code, which resulted in a separate summary matrix for the three groups of young people and parents’. This allowed for comparison, exploration and explanation of patterns emerging [42, 46]. For the purpose of this paper, themes related to information during the transition process were extracted from each of the summary matrices. Themes and subthemes were then synthesised across the four groups of participants.

## Results

A total of 64 young people and 28 parents were interviewed from 10 NHS Trusts across England. As Table 1 illustrates, we successfully recruited some young women with ADHD of each age group, but only three fathers. The sample is described in greater depth in the NIHR CATCh-uS report [40].

**Table 1:** Transition stage, gender and age-range of participants

Stage	Gender			Age-range	Total
	M	F	F&M		
Pre transition	16	5	–	14–17	21
At transition	13	9	–	17–21	22
No transition (re-entered as adult)	15	6	–	19–29	21
Parent	1	25	2^a^	N/A	28
Total	45	45	2^a^	14–29	92

Notes: *M* Male, *F* Female, *F&M* Female and Male, ^a^both parents present, and interviewed together

Three themes emerged relating to the role of information in young people’s experiences of transition into adult ADHD services, their transition outcomes, and their confidence in their ability to self-manage their condition:

1. Navigating information with help from parents
2. Information on ADHD into adulthood
3. Information about the transition process

The first theme referred to the essential role of the parent in navigating information, while the two others suggest that two distinct types of information may be necessary for a smooth transition. Interviewees reported a range of experiences, however a reliance on parents to gather and translate key information, and difficulties as well as negative emotional experiences associated with poor quality communication of information, were universally reported. These are illustrated below with sub-themes. For a summary see Table 2.

**Table 2:** Descriptive themes and sub-themes, with stages relative to transition at which they emerged

Theme	Stage ^a^	Sub-theme
Navigating information with help from parents		**Parent**
	0,1,P	Translates treatment experiences; interpreting clinical advice for young person.
	0,1,X,P	Retains informed overview of ADHD as a condition; holds understanding of young person’s potential long term treatment needs often when young person cannot; can guide treatment decision.
	0,1,X,P	Persistently seeks service information necessary to continue access to care.
	0,1,P	Navigates and manages administrative information on behalf of young person; for example helping record appointment dates, and locate service addresses.
	X,P	Attempts to access information and signposting to services through General Practitioner (GP); often without success.
	1,X,P	Coaches/supports young person in navigation of administrative information; helping them practice information management strategies.
	0,P	Role effectiveness limited by parent’s understanding of ADHD and knowledge of service provision.
		**Young person**
	X	Seeking information necessary to access care is impossibly difficult.
	X	When asked GPs do not provide appropriate information.
	X	Communicating with adult services is difficult and distressing.
Information on ADHD into adulthood	0,1,P	Informed will definitely grow out of ADHD; unhelpful and inaccurate.
	0	Limited information provided about condition; good start but many want to know more.
	0,1,P	No information given.
	1,X	Told might/might not grow out of ADHD; starts process of self-reflection.
	1,P	Understands long term support may be needed; thinks about future care.
	1,P	Sufficient information provided; develops a nuanced understanding of long term care needs.
	0,X,P	Wants information about ADHD as a condition to come from experts (clinicians); relying on parent not sufficient.
Information about the transition process	0,1,X,P	No transition information provided; one young person did not mind, many felt left ‘*in the dark*’.
	0	Basic information provided, that transition may happen.
	0,1,X,P	Insufficiently detailed information provided to enable young person to prepare for transition.
	1	Sufficient information provided; emotional comfort and confidence in transition process.
	0,1,X,P	Relies on information from informal sources; often worrying, which causes distress.
	1,X	No contact point for information during transition, or when out of services; highly distressing.
	X,P	No information on how to re-enter services as adult; a barrier to accessing care.
	1,X,P	GPs are key point of contact; but inconsistent and confusing sources of information, leading to difficulties accessing care and emotional distress.

**Fig. 1:**
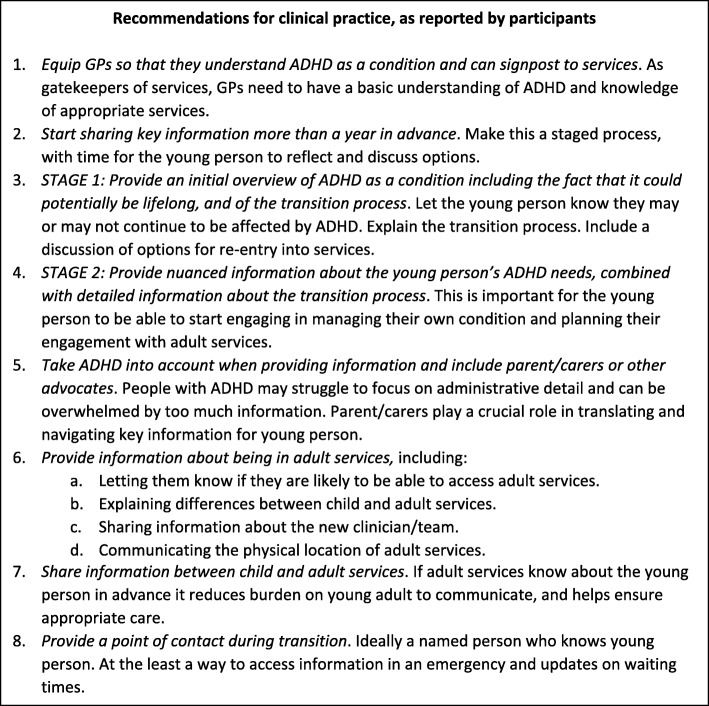
Recommendations for clinical practice for all health care practitioners supporting young people with ADHD; child and adult service clinicians and GPs (for supporting quotes see Additional file 1)

Recommendations for clinical practice were made by interviewees, these are presented in Fig. 1 with supporting material in Additional file 2. They included:

- *when* to start communicating about transition,
- *what* information to share, and
- *ways* of communicating relevant information.

### Navigating information with help from parent

#### Parent essential

Having a parent to seek, navigate and translate information about *ADHD into adulthood*, and *transition between services* was a crucial aspect of service engagement for the majority of young people. The term ‘navigate’ encapsulates the way parents steer the young person through transition, by finding and helping them process the information they need. Without such advocacy, many would not have transitioned.

“If I didn’t have the parents that I did, I’m scared to imagine where I would’ve ended up because it’s not good. It’s not good at all.” F-1

“[Without mum] I’d have no clue. I wouldn’t have known about the medication types, I wouldn’t have known that there was an adult services I could go to and I wouldn’t have done it myself” M-1

The reliance on a parent for support was not defined by chronological age. Young adults also reported needing the support of a parent/carer to find and manage information needed to re-access care as adults.

“All I can remember is my support worker from my hostel taking me there… I didn’t know where to go and I think she helped me, pushed me in the right direction, yes she did that.” M-X

#### Persistently seeking information

In the majority of cases, the information on *ADHD into adulthood* and *on transition processes* needed for a young person to transition into adult services was not readily available, and parents demonstrated incredible persistence in their attempts to access it.

“From the age of 17 I was on the paediatrician’s back saying, “Look, what happens next? Can you refer us to adult ADHD?” …So I found all the information out, I went to my paediatrician with that and said, “Look, can you refer us to this?” F-P

Many parents approached GPs for information, but for the majority this was not a successful strategy.

“I think mental health services are one of the areas that possibly GPs don't know enough about.” F-P

“It should be easier for parents with their GPs, so there should be more information for GPs and an easier referral system…so that there’s a degree of support there from the beginning before the actual disillusionment and the stress and all the rest of it and the isolation,” F&M-P

#### Teaching information management

A few parents described coaching young people in the development of administration and information management skills needed to gradually increase self-management.

“I do what I can. I sit and help him fill in the application forms, I talk to him about it.” F-P

“I am helping X become an adult… I’m moving him secretly into his life.” M-P

#### No information support; no transition

Those young adults that had not transitioned appeared not to have received sufficient information pre transition, and reported multiple failed attempts to access information about treatment in adult services.

“I don’t think there’s really a straightforward route people can go and find out for ADHD.” M-X

“I’ve been begging for help for absolute f***ing years.” M-X

Some reported chaotic service structures that made maintaining contact or finding out what was happening incredibly difficult, leading to a sense of failure and experiences of emotional distress.

“Every time I ring I never get through to anyone. ...it seems to go around loads of different offices. I ring up someone and I say, ‘Oh, can you check?’ and they say, ‘You’re not part of our department. We’ll put you through to another one…It’s getting me down because I want to know where I’m going, what direction I’m going in and I’m getting mixed signals from both different professionals….It got me worried.” M-X

Without support from a parent, these young people were confused by complex systems, could not stay engaged with services, and were therefore unable to access treatment.

“I rang them up to tell them that I’ve moved ... and just got voicemail after voicemail. I left a voicemail and I still haven’t heard back.” F-X

### Information on ADHD as a lifelong condition

For all young people and parents’ a basic understanding that ADHD might continue into adulthood appeared to be related to understanding the need to remain engaged with services. Developing a more nuanced (or detailed) understanding of ways ADHD symptoms might change with age appeared to be related to a reflective approach to self-management of symptoms into future, for example through learning and applying strategies.

#### Pre transition

Before transition, although registered with a health service and receiving treatment for their ADHD, young people and their parents reported very little understanding of ADHD as a lifelong condition and a distinct lack of information on what this might mean for the young person as they entered adulthood.

“Don’t know what to expect, haven’t thought about it.” M-0

“I don't know. It just remains to be seen.” F-P

In some cases they had been given the misleading message that ADHD would definitely not be an issue into adulthood.

“We’ve been under the impression that ADHD is not recognised as an adult problem in this area. Like they're supposed to grow out of it at 18.” M-0-(P)

In the context of being interviewed about transition, some young people and parents expressed a desire to understand more about *ADHD into adulthood*. They believed this would help them to prepare for the future.

“I think it would be useful if … they were spoken to by someone … saying, ‘You are 16 now, there’s lots of options that you can do… to have someone who knows about ADHD to say, ‘these are your options.” F-0-(P)

Some young people wanted to know more about ADHD to help them to develop more insight into their condition.

“For me it’s all about finding out why I have it, what it actually is and what can be done to change it in the future.” “It’s nice to almost have that… insight.” F-0

“I feel like if I was briefed on what knowledge people have about ADHD and why I have it … I’d feel a lot more comfortable.” M-0

They wished to learn from people with expert knowledge.

“He would like a relationship with someone who he can ask these questions of …consistency with a knowledgeable, qualified person that can actually help him move into adulthood.” M-0-(P)

#### At transition

For those young people at transition whose clinicians had not discussed *ADHD into adulthood*, this uncertainty was linked to significant distress and confusion about how they would manage without treatment.

“I said because I'm going to uni hopefully, I probably want to stay on the medication. So yeah but then in the Child's place they were talking about taking me off it. …I don't know how it's going to happen? I'm just like going to be taken off of it at some point and then expected to be able to move forward without doing anything else? I don't really know. But no they haven't really said anything about that.” F-1

By contrast, those that had been provided with the basic information by their clinician that their ADHD might continue to affect them into adulthood, had some limited understanding about potential future treatment needs.

“So it could get worse or it can ease off and you tend to grow out of it. But I don’t think I’m going to grow out of mine. I can’t see it happening.” M-1

The more detailed information young people had about their ADHD and ways it might change, the more self-reflective and nuanced their discussion of future needs appeared to be, including consideration of ways they might self-manage their condition.

“I know that it’s not going to be a quick fix. … … I want them to give me the tools and I’ll build the house. I don’t expect them to do it for me, but...” F-1

“She assessed me before I finished and just said my ADHD has calmed down from when I was little and it will probably calm down a bit more when I get older, but I will still always be that hyperactive child… she [clinician] said as you get older I will start to recognise signs more and learn how to control it.” F-1

Developing a nuanced understanding of ADHD as a condition, including the fact that symptoms may change over time; that environments such as jobs or studying may interact with their symptoms; and that use of self-management strategies can improve with maturity, may promote positive attitudes to self-management as well as help seeking in the young person and parent.

“I’d say information for empowerment, definitely, is what adults need around ADHD, and teenagers especially so they can know their condition.” M-X

#### No transition

The majority of young adults who had not transitioned had received inaccurate or no information about *ADHD into adulthood*.

“I thought you just grew out of it. And so there was no information about being an adult with ADHD, like jobs might be hard sometimes and stuff like that.” M-X

Looking back, they expressed anger, regret and distress about the information they received as teenagers.

“I feel bad speaking ill of my psychiatrist there because he did help me … but he did leave me under the impression that I wouldn’t have any more issues with ADHD and it’s not something that I’d need to be… I’d need to see in the service anymore….I felt there was misinformation, yes.” M-X

“Even medical professionals, they all told me, ‘It will go away when you’re about 17 or 18 and you’ll be a normal person.’…It’s the biggest crock I have ever heard.” M-X

Several parents reported that their GPs’ lack of understanding about ADHD was a barrier to accessing treatment or support.

“I think that’s critical. That’s your first point-of-call, I would have thought.... She just said, ‘Tough love.’ I’ll never forget that.” F-P

“My GP has absolutely no idea about anything. He's been telling me and X his whole life there's nothing wrong with him. So I don't know how I would re-access those services.” F-P

### Information about the transition process

#### Pre transition

The majority of young people approaching transition reported that they had been provided either with very limited or no information about the process. They did not know if or when transition might occur, what processes were involved or what to expect of adult services. While some of the youngest in this group were not concerned at this lack of detail:

“It won't affect me, I'd probably still carry on.” M-0

By age 16 the majority wished to know about the transition process in advance so that they had time to plan and prepare themselves for change.

“I’d have to know what they’d even do to be able to ask questions.” F-0

“I’d rather have time to plan…I don’t like sudden changes.” F-0

“Instead of just being left in the dark after you’ve left the child stuff.” M-0

“I’d rather it sooner than later because then I know what to expect …because it's my future and I don't really know what's going to happen.” M-0

Some of the eldest pre-transition interviewees expressed anxiety about the lack of information on services for adults, fearing their symptoms might become worse if they did not have health service support.

“I kind of worry that it’s going to get to a point where I won’t know where to go…it will just get worse and the cycle will start.” F-0

Parents of young people pre-transition stated a need for clear information about available services, which was not met.

“A list of all the services and how to access some would be really good. Also not just services to do with his medical needs but also the wider things.” F-P

In their role supporting young people, parents (some of whom heard about transition for the first time from the interviewer) explained that they needed to know about transition in advance to make plans, prepare the young person for change, and help them manage their anxiety.

“Having someone say before he got to 18, saying ‘Look you know it’s going to end when he’s 18, the services here, you'll then need to go to…’I could have had something in place or you know but I weren’t’actually given that opportunity.” F-P

“Yeah it's being informed. If you're informed then you've got like ahead of the game if you know what I mean? You feel like you can prepare the ground ready for them to come into it. But as I am now…I'm like, I don't know nothing X, and that obviously with anxiety kicking in that's not good.” F-P

Faced with a lack of information about the transition process, some parents relied on informal sources of information. In these cases, reports of poor adult services and transition failures tended to increase their concerns.

“I am dreading him going, changing over services, because I know what mental health services in X are and I know how awful they are at the moment and I know there is hardly any.” F-P

“It scares me from people that I've known…it makes me nervous as to what will happen when he comes out of Children's Services.” F-P

### At transition

Several young people already in the process of transition still had no information about where they were going after reaching the age boundary for child services, or what would happen.

“[I] kept ringing the numbers to see where I was going, but no one would ever answer.” F-1

“Not a clue. I don't know even if there is a building.” F-1

Having no point of contact or named person to advise them on simple procedural matters, such as the address of a service, or time remaining to wait for an appointment, caused intense distress. For many this also had an impact on concurrent life events such as exams.

“panic stations and not coping with the thought of being 18… As it is now, I don't even know where it is. I don’t even know the name of it. So I know nothing.” F-P

“when I called they juggled me round departments for ages and then realised that they hadn’t even sent the letter out, so after two weeks in exam season of not having any clue, I was then told that they hadn’t done anything…” M-1

The majority of young people at transition reported not knowing what to expect from adult services which meant they felt unable to prepare themselves. This affected their first experiences of adult services.

“It was a bit intimidating…having the information first we would have understood what the place was, but when we first walked in we had no idea…” M-1

“It doesn’t help because I get mega anxious and nervous about new places and not knowing anything about a new place in the first place is…” M-1

Some reported that their child clinician did not appear to know enough about local adult services to be able to answer their queries.

“I wanted to know how adults was but she didn’t know, that’s the truth. No one in CAMHS seems to know what adults is actually like.” F-1

Not knowing what to expect of adult services was a major cause of distress, so that even if there was a ‘successful’ transition, the anticipated experience was emotionally stressful and negative. One young person explained how their ADHD affected the way they experience new environments, and why not knowing anything about adult services in advance was so stressful.

“I describe ADHD as trying to pay attention to everything at the same time. And if you're doing that in a completely new place where you don't know anything, that's exceptionally overwhelming, I completely shut down, I feel incapable… I don't know what their job title is let alone what they look like, let alone their name, let alone what they're like and it terrifies me. When I go in it's going to be awful.” F-1

A few young people in transition reported receiving sufficiently detailed information in advance and described the transition experience positively, in clear contrast to the majority of interviewees.

“Yeah, good. They prepared me. They told me all the information, what I needed to know, told me I was moving. They informed me quite a lot.” M-1

Information about the expected transition from the child clinician, provided early, and repeated over a period of time, appeared to provide a sense of stability at transition stage.

“She’s always told me about the adult clinic. From a young age she’d tell me that once I get to a certain age I’ll have to leave her and I’m going to have to go to the adult clinic...amazing doctor.” F-1

Even if the information was that there was no service, it seemed that knowing in advance was better than finding out when they had already left child services.

“My consultant told me that I probably wouldn’t qualify for the adult psychiatry…at the moment I’m kind of like okay, but I think… when it was first mentioned I was completely distraught.” F-1

### No transition

Young adults who did not transition reported having received no information in advance about the transition process.

“There was no discussion about that. I didn't get told about anything, I didn't know there was any support for adults.” M-X

“No paediatrics are aware of it being an adult thing…they are not referring people on to the ADHD adult services for help.” F-X

Those that had not transitioned, reported that finding out how to re-enter services was particularly difficult. Several believed that it would have been helpful to receive this information before they left children’s services.

“What might have helped because we decided to drop out, if they gave us some information for later on in life in adulthood.” F-X

“There was never any discussion about if you do need post 16 care this is where to go, this is how to apply. Nothing like that.” M-X

For many young adults who had lost contact with child services, one strategy for information seeking involved asking GPs for support. When GPs were well informed, or searched for information on the young person’s behalf, this was helpful.

“Some of them, they really know their stuff but then … two GPs four streets away don’t have a clue. So its consistency, I think.” F-P

However, many reported that their GP did not know about suitable adult services, and therefore provided no support and/or inaccurate information.

“It would be better if the GP actually knew who to refer you to.” F-X

“You should at least go to your GP and be like ‘Could I go back on this [medication]?’they say ‘Of course’you know like I need support well here you go…But I didn’t even know about that at all. I wasn’t given that information.” M-X

For many of these young adults, the gap in care they experienced was seen by their parents as linked to serious life issues they had faced, such as problems with the police.

“If we’d have accessed it when he was 18 I still believe to this day he wouldn’t have been in the trouble he was in.” F-P

“So at the point of discharge he was just coming up to his 18th birthday …from then is when everything started unravelling for him.” F-P

[Note: F = female, M = male. 0 = pre-transition; 1 = at-transition; X = no-transition (re-entered as adult). P = parent. (P) = Parent commenting within young person’s interview.]

## Discussion

Our findings demonstrate that communication of key information on *ADHD into adulthood* and *transition processes*, well in advance of transition in a manner that takes account of the *essential role of parents*, is important for a successful and positive transition experience. The majority of participants reported experiences of poor information communication, associated with emotional distress and difficulties in the transfer of care. These findings support and extend recommendations made in the NICE guidelines for treatment and management of young people with ADHD [3, 11]. Recent research has highlighted that relatively few young people with ongoing treatment needs related to their ADHD successfully transition, with those that do experiencing low quality transitions that fail to adhere to the NICE guidelines [15]. In particular, poor provision of information about adult services (to patients and clinicians) has been identified as a barrier to transition [30]. Our findings provide key evidence on the role that information provision may play in these transition difficulties.

### Navigating information with help from parent

Participants with ADHD reported needing help from a parent to gather, navigate and manage the information necessary to access services; a need that continued into adulthood for all participants. While the need for continued parent/carer involvement in communication has been recognised across long term conditions [47], these findings provide evidence of specific needs of young people with ADHD, which are likely to be different due to the nature of ADHD to those already recognised for young people with other long term conditions such as diabetes or eating disorders [11, 47]. This finding ties in with findings by Colver at al [48]. that young people with neurodevelopmental disorders, are in a less independent phase at transition compared with those with physical conditions such as diabetes mellitus. This is likely to be because of the key symptoms of ADHD that have implications for processing and managing information, and evidence of delays in brain maturation associated with ADHD [49, 50]. At transition, the information processing load is likely to be higher, due to the need to take on new information, manage changing processes and prepare for new experiences, therefore provision of key information in simple clear formats and via several methods, to both the young person and their parent becomes crucial to support transition.

Not including parents in communication of information was reported as a barrier to young people’s engagement with services in our study, as well as in previous work [25, 38]. In contrast to the family orientated approach of child services, AMHS tend to adopt an individual patient centred approach which can lead to parents feeling or being ‘cut out’ [51]. This cultural emphasis is in conflict with the reality that many young adults with complex conditions report needing a degree of parental involvement in adult services [52]. A recent study of transitions for young people with a range of long term conditions found that ‘appropriate parent involvement’ was one of three service features associated with better outcomes for young people [48]. In line with the NICE guidelines on transition [11], adult services need to offer an appropriate level of care for young people with ADHD, taking into account developmental maturity. Findings from this study indicate that planned inclusion of parents in communication of key information, with consent from the young adult, is likely to be required for continuation of care in the case of ADHD.

### Information about ADHD into adulthood

In order to engage with adult services, the young person needs to understand that ADHD can be a long term condition [53]. For many participants this basic information was either not being communicated or was not understood. In line with previous research, young adults that had not transitioned reported being ‘misinformed’ that adult ADHD did not exist [31, 33, 34, 39]. Many younger participants ‘at’ or ‘pre’ transition, had not received information about ADHD as a potentially lifelong condition, meaning that despite an increasing awareness of the prevalence of adult ADHD [54, 55] poor communication of this remains an issue in clinical practice. If young people and their parent/carers believe (or are told) that ADHD is something that they will definitely grow out of, then they will not have the essential facts that they need when considering the transition process. They may assume that they will not need treatment once they have left school and therefore not prepare for transition or pay attention to relevant information. Lack of communication about the potential persistence of ADHD into adulthood, more than a decade since this knowledge has been written into clinical guidelines [2, 56], is a service delivery issue that could be addressed at relatively little cost.

The NICE guidelines on patient experience in adult NHS services [57] clearly describe the importance of communication and provision of appropriate information in enabling patients to actively participate in their care. The need for condition specific information to support transition has been recognised for other long term conditions such as diabetes and eating disorders [47, 58], and our findings demonstrate that this is particularly important in the case of ADHD, especially given the relatively recent recognition of ADHD as a long term condition. Self-management of chronic conditions often relies on effective communication of high quality information [26, 27] and promotion of health self-efficacy has been evidenced as an important factor in supporting transitions for a range of long term and complex health conditions [48, 59]. In line with this research, our findings indicate that the development of a nuanced (or more detailed) understanding of ADHD pre-transition may facilitate a process of reflection by young people on self-management of their condition so that they can better understand their healthcare needs into adulthood. Several participants wanted information on ADHD to be provided by ‘experts’. While clinicians working in child services have a role in such provision, alternative ‘expert’ sources such as adult ADHD service clinicians, peers with lived experience, and/or specialist ADHD nurses should be considered [47, 60].

### Information about the transition process

NICE guidelines recommend provision of practical information about transition processes and adult services as young people approach transition [3, 11]. The majority of participants could not recall receiving sufficiently detailed information; lack of information was thought to contribute to failure to transition, and was a cause of emotional distress, which replicates existing evidence that poor information provision may be a barrier to continued access to services in complex care [30, 61].

It is possible that transition information is not provided because clinicians do not know what is available, or are not familiar with eligibility criteria in adult services [22, 30, 62]. In the ‘Transition from CAMHS to AMHS’ project, failure to transition was more often related to failure to refer, as rejection was assumed, rather than because adult services declined referrals made [4]. Transition preparation might also be neglected due to workload and resource limitations. Communication of appropriate information on the transition process relies on the availability of accessible and acceptable adult services. Until recently there has only been limited information on availability of adult ADHD services in the UK [17, 18, 63]. However, a national survey conducted in 2018 as a part of the CATCh-uS study [41] has resulted in creation of a map of services informed by over 2500 by service users, health workers and commissioners, available via the study website [64] and the UK Adult ADHD Network [65]. Providing this level of national data on service provision, in line with recommendations from NHS England’s Five Year Forward View for mental health [66], may facilitate better communication of information about service availability in future.

### Strengths and limitations

These data were an analysis from a large qualitative sample, recruited from 10 NHS trusts in different areas, and with different levels of adult ADHD service provisions. We used a sampling grid with an interim analysis to ensure that as broad range of experiences were represented and interviews were conducted using a topic guide that was revised and refocused at the interim analysis. Therefore, we are confident that our data reflects a broad range of lived experience of ADHD into early adulthood.

Recruiting participants via NHS trusts has restrictions for the sampling pool. According to NHS ethic’s restrictions only young people registered with a service at the time of recruitment were allowed to be approached for participation in this study. Therefore, young adults who had received treatment for ADHD at the selected trusts, but had disengaged with services (and thus at the time of the study were not officially registered as patients) could not be invited to partake in the study. We know little about the experiences of adults with childhood treatment of ADHD who fail to transition and remain out of contact with services In future, research needs to involve young people who are not currently registered with a service, in order to hear their perspectives on the role of information in transition.

### Implications

Evaluation is required to establish the most effective methods for information provision to young people with ADHD and their families, without adding to the workload of already overburdened health professionals. Future research needs to explore ethical issues faced by adult services in balancing patient confidentiality against a need for long term advocacy for young adults who are developmentally predisposed to struggle with managing their long term condition [25, 67]. These issues need urgent resolution so that the healthcare needs of young people with ADHD can continue to be jointly managed between young people, their parent/carers, and their clinicians into adulthood [68].

As gatekeepers of services, GPs play an essential part in communicating key information to young people with ADHD and enabling to access continued care. However, GPs may feel insufficiently equipped to do this, both in terms of knowledge about ADHD, and in terms of being able to signpost to appropriate services [20]. There is consequently a need for evaluation of the best ways to support primary care practitioners in this role, and to understand how shared care arrangements, (defined as the planned joint participation of consultants and GPs in the delivery of care for patients with a chronic condition [69]), can optimise communication between all providers involved in transition [70, 71]. Findings indicate a need for improved medical education for GPs and other health workers, such as specialist nurses and practitioners working in AMHS, to facilitate continuity of care. Commissioners and providers also need to produce clear information about local services for adults with ADHD, their remit, and referral criteria, to enable GPs to effectively signpost and refer young people for support.

Currently, there is a great deal of variation in configuration of services for adult ADHD, with some controversy around optimum models of care [70]. Consistent national implementation of transition guidelines with a clear clinical pathway into adult care for young people with ADHD would facilitate information transfer for clinicians. It would also allow for universal resources about the transition process, such as leaflets and short videos to be produced nationally (and made available via websites and apps), forming a cost-effective information resource for both patients and health care providers. A recent national survey mapping adult ADHD services in the UK has to some extent addressed the lack of information on locations of current services [41, 64, 65]. This may impact positively on young people with ADHD who are trying to find an adult service to transition into, help clinicians to access the information they need to advise young people on where to go for an adult service, and support commissioners to make informed decisions about where investment is needed.

In the current economic climate, and with constraints on NHS provision, potential areas for service development informed by this research could include; supporting services in the communication of key information on ADHD as a long term condition through developing national materials, and highlighting the locations of current adult services so that information about these is readily available. Developing a national information resource on living with ADHD as a condition, and what this may mean for young people, in a format that is developmentally and cognitively appropriate for young people with ADHD and their parent/carers could provide a tool for service managers to support transitions. ADHD is often diagnosed in early to mid-childhood and may present differently over the lifespan [72]. It is therefore important that clinicians revisit diagnosis and prognosis with the young person and their carer as they mature. Services should implement annual reviews for patients to discuss how ADHD is currently affecting key areas of their life, and the potential impact of their condition on their life into the future.

Many young adults interviewed had inspiring and relevant stories of their own transition experiences to share. Developing a mechanism to share stories of ‘experts’ with lived experience of an ADHD transition, in a way that was accessible to those just starting the transition process is one method through which information could be shared [47]. One possible approach could build on research carried out by Coyne et al. [73] with adolescents and young adults with long term illnesses such as heart disease and diabetes. In this project, participatory methods were used to co-design information and a website, in order to support young people in their transition to adult healthcare [73]. Young people expressed preferences for information that was trustworthy, empowering and available online. They also desired video testimonials of experiences from young adults that had already transitioned [73]. Given that stories can increase the quality of medical decisions, and improve health judgements [74], future research could use a structured framework of participatory design to develop informational resources designed to meet ADHD specific needs [75].

There is a need to make use of available technology to share information with people who use and deliver services [76]. Providing information digitally could be a scalable and economically viable way of supporting stretched services to provide relevant information about transition. Technology based interventions, which involve the use of equipment such as mobile phones to enhance care through improved communication and enhanced ability to process information, have been used to support patients with other long term health conditions such as heart disease and diabetes, but there is only mixed evidence on their effectiveness [77]. However the use of narrative stories, including stories of peer experiences, has been successful in improving a range of health outcomes related to self-management of long term conditions such as hypertension and diabetes [78–80]. Information could be shared effectively with patients with ADHD using digital and technological solutions.

## Conclusion

This study provides insight into how the appropriate communication of information can contribute to a successful transition and support a young person in their journey towards self-management. Many of these findings are likely to be generalisable to young people with any long term condition, such as diabetes or eating disorders [47]. However specific to the ADHD population are the difficulties caused by variable service models/lack of adult services, and the lack of understanding about ADHD as a long term condition. Involving parents/carers in the provision of healthcare into adulthood optimises the chances of continuity of care. Making information about ADHD and transitions available on the internet and social media could address gaps in information provision identified in this research while managing the workload limitations of existing services. A role for peer education programs, led by young adults with ADHD is indicated. Improving information provision is likely to have a positive impact on transition outcomes, however addressing current structural barriers to transition caused by patchy provision of adult services remains an urgent priority. Understanding and, where possible, addressing barriers to appropriate communication of information to this group will be an important step in facilitating transition.

## Supplementary information

**Additional file 1.** Interview topic guides.

**Additional file 2.** Recommendations for clinical practice, with supporting quotes.

## Data Availability

The datasets generated and/or analysed during the current study are not publicly available as they are under embargo until the end of the CATCh-uS project (2019), but are available from the corresponding author on reasonable request. Data is currently stored securely by the University of Exeter College of Medicine and Health.

## Abbreviations

ADHD: Attention Deficit Hyperactivity Disorder
CATCh-uS: ‘Young people with ADHD in transition from children’s services to adult services’ study
GPs: General Practitioners
NHS: National Health Service
NICE: National Institute for Health and Care Excellence
NIHR: National Institute for Health Research
UK: United Kingdom
