# Supplementary material for: In transition with ADHD: the role of information, in facilitating or impeding young people’s transition into adult services
Source: BMC Psychiatry. 2019 Dec 17;19:404. doi: 10.1186/s12888-019-2284-3 (PMC6918680; doi:10.1186/s12888-019-2284-3)
Supplement: Supplementary file 1 — Additional file 1. Interview topic guides. [file 12888_2019_2284_MOESM1_ESM.docx]

## Interview topic guides

### Topic guide 1: young people pre-transition

1. **Current contact with services**

- Do you remember when you had your last appointment at CAMHS? And can you tell us a bit about how that went?
  - Who was present? (who was there – parent, clinician, nurse)
  - What was the appointment for? (Prescription, talking about transition, medical review, discuss other problems, did you discuss other problems, such as anxiety, depression…)
  - Where you asked questions? Could you answer for yourself?
  - Did you have questions for your clinician / nurse? Did you get to ask them? (Why not?)
  - When you left the doctor’s consultation room, did you understand why you had attended the appointment, where any decisions made, was anything going to happen / change as a result of the appointment? (ATTENTION: we want to know what the YP perceives! Not what actually happened, no point mum adding to the conversation now)
  - Looking back, and what you have just told me, do you feel like you were a part of what has been said and or decided?
    - Did you feel your contribution made a difference?
    - Did you discuss what happened at the appointment with your mum on the way back?
    - Did you feel like it was worth going? (Why not?)

1. **Managing your care**

- How much would you say, your mum helps you with managing your ADHD? (remind you to take medication, order prescriptions, make new appointment, attend the appointment, drive there, asks questions at the appointment about your medication …)
- Which of these things do you think you will soon be able to do yourself?
  - What do you think would help you to be more confident to do these things yourself?
  - Has anyone suggested you do these things yourself (and if so, did you try and what happened?)

1. **ADHD as long-term condition**

- What is the main reason that you still attend CAMHS? (What is currently the main reason to continue your treatment?)
- Do you see yourself going to CAMHS or any other services for your ADHD in the future? (Suggestion: at 16/18, when leaving school …)? Do you think you will still need help or support for your ADHD after you leave school?
  - Why (not)?
  - What would be a reason for you to stop attending CAMHS or receiving help for managing your ADHD? Could you see a reason at the moment to stop going to CAMHS?
  - What do you think would happen if you stopped going (do you think you could go back after a few years?) would you want someone to keep contacting you to see how you are doing? Would you want someone to encourage / persuade you to stay / attend appointments? How do you think your family / mum would react?
- Do you think ADHD may still be a problem at 18, 20?
  Do you think at some point some of the symptoms might be more difficult to deal with and you might need support again? (hence, you would return to services)
  Who would you turn to for help if you found out you were still struggling with things related to your ADHD?
  - GP – do you ever see your GP? Do you get any help from them with your ADHD?
  - If you ever go to your GP for something, do you get to talk about your ADHD as well? / does the GP ask about your ADHD?

1. **Transition**

- So, has anyone talked to you about that? (That you might have difficulties later on in life; or that you might need support after you have stopped going to CAMHS)
  - Has transition been discussed at one of your CAMHS appointments?
    - Do you know until what age you can attend your current CAMHS?
    - Do you know where they will refer you after 18?
    - Has anyone talked to you how this service might be different from your current CAMHS?
  - Is there anything specific that you would want to know and how would this help you? Is there anything specific that you are worried about (related to your ADHD when you get too old for CAMHS services)?
  - Is there anything you have been asked to do to prepare yourself for the next step, or for going to this new service?

1. **Closing question**

If you could ask your clinician just one question about the next step after leaving CAMHS / reaching 18 (might be adult services, might be dealing with life without any support), what would it be?

If you could ask your mum just one question about the next step after leaving CAMHS / reaching 18 (might be adult services, might be dealing with life without any support), what would it be?

***Add-ons for Young person in residential school / care***

Who helps you with managing your care?

Who goes with you when you meet your clinician?

### Topic guide 2: young people at transition

**1. AMHS**

- When was your last appointment with AMHS?
- Can you tell me a bit about what was that like?
- Do you have any co-existing mental health conditions alongside your ADHD?
  - If so, were these discussed in your appointment with AMHS?
  - Do you any support or treatment elsewhere for these?
- Was your appointment with AMHS different from CAMHS?
  - If so, in what way?

**2. Transition**

- When did you first realise you might need support after leaving CAMHS/turning 18?
  - (If they realised it themselves, did they ask their clinician/anyone else about this? If not, why not?)
  - What sort of support did you think you needed?
  - How did you feel about still needing support?
- When was transfer to AMHS first discussed during a consultation/with CAMHS?
- What was discussed with you?
  - Were there things you wanted to ask but didn’t ask? (Unanswered questions?)
- What happened next?
- Was there a planning meeting? (Who was there?)
- Did you meet anyone from AMHS before your first appointment there?
- Can you give a few details about how the adult service was in contact with you?
  - Did you get a letter? Who got it, when, did you see it?
- How long did you have to wait to be seen by AMHS?
- Whilst waiting, what did you know about what would happen next?
- Whilst waiting, what happened to your medication in that time?
- Was it clear who you could contact for help throughout the process?
- Could you still contact your old CAMHS service? Did you have a named contact or transition worker at CAMHS?
- Did you have a named contact or transition worker at AMHS?
- Were there any gaps in your care when you moved from CAMHS to the service for adults, or times when you could not get the help you needed?
  - If so, what happened during that time?
  - Did you know what was happening or who to contact?
  - Did you contact or try to contact anyone?
- At your first appointment with AMHS, did you know who you were going to see?
- At your first appointment with AMHS, did you know where to go?
- What did you think about how CAMHS and the service for adults worked together?

**3. Role of the GP**

- How has your GP been involved in your ADHD care so far?
- Would you get in touch with them if you needed help? (Why/ why not?)
- Do you think that there is anything that your GP could help you with?

**4. Managing as an adult**

- Now that you have taken the next step into AMHS, do you feel confident managing your care? (e.g. making appointments, ordering prescriptions, getting to appointments)
  - (*If they mention their mum does all of this -* if it wasn’t your mum doing this for you, would you be able to do it yourself? Would you know what to do?)
- Do you think you’ll become more confident managing your care in future?
- What do you think would help you get more confident/manage better?
- What do you think your CAMHS team could have done to prepare you for managing your own care as an adult?
- Is there anything specific that you find difficult about managing as an adult with ADHD?
- Do you think there is support out there to help you?
- What sort of support do you think would help?

**5. Giving advice and closing interview**

- - If you spoke to a younger person who is still in CAMHS with ADHD, what would you tell them to expect as they get older?
  - What advice would you give them about treatment, symptoms and medication?
  - What advice would you give them about moving to adult services?

### Topic guide 3: young adults no transition

1. **Returning to services**

- What triggered you to go back into services?
  - What influenced this decision to go back at this point?
  - Did you discuss this with anyone?
  - Was this the decisive moment?
  - Whose idea was it to go back into services?
- What happened next?
  - Can you describe the steps you took?
  - Who did you go to first?
  - Did you still contact your old/previous doctor? What happened?
  - How did you get referred to the service you are attending now?
  - Were there any difficulties in getting seen by adult services?
  - Did you get a letter? Who got it, when, did you see it?
  - Did you have a contact number or a named person?
  - Did you/your parent ring them?
  - How long did you have to wait?
  - If you were waiting, what did you know at that point about AMHS
  - How did you feel about this process?
- **Do you have any co-existing mental health problems?**
- Did you get a chance to discuss your mental health more widely with the person who referred you to AMHS?
- When you got to AMHS, did you discuss your mental health in general?

**2. Leaving services**

- Can you tell me a bit about when you stopped going to CAMHS and why that happened?
  - Did you discuss this with anyone?
  - *(if they stopped taking medication) -* Did anyone discuss what might happen when you stopped medication?
    - Did anyone discuss how that might affect you?
  - *(If they disengaged)* – did anyone ask you why?
    - Did anyone try to stop you?
  - *(if was their choice to stop going*) Was there anything that would have made you change your mind? (E.g. decide to keep taking medication / stay at that service / transfer to adult services?)
  - When you left, did you feel you could come back?

**3. Current care**

- Has your GP been involved since you left CAMHS services?
  - If not, why was that?
  - If yes, when were they involved? What did they do?
- Now you’re in AMHS, do you feel confident managing your care?
- Is there anything specific that you find difficult about managing as an adult with ADHD?
- How are you coping with these difficulties?

**4. Reflection/Close**

- If you spoke to a young person who was about to leave CAMHS, what advice would you give to them?
  - What advice would you give them about medication?
  - What advice would you give them about moving to adult services?
  - If they want to leave services, would you encourage them to stay? Why/or why not?

### Topic guide 4: parents/carers

1. **Current situation**

- How old is your child?
- Could you tell us a little bit about any help your child receives for their ADHD?
- How you involved in the treatment of your child’s ADHD?
- Does your child have any (comorbid) conditions as well as ADHD?

1. **“Were you/your child discharged from services if you missed appointments, even though you/they still had an ongoing need for support?”** “What happened then?”
2. **What would happen/has happened if you chose not to use medication but still want to be in touch with services?** Can you do this?
3. **Future (Transition)**

- If currently, your child takes medication for their ADHD. Do you see this continued in the future?
- How do you see this future support?
- What are your thoughts on ADHD and growing up (how this may present into teenage and adulthood)?
- What are your thoughts on medication for ADHD, what about medication into adulthood?

1. **Planning and preparation for transition**

- When did your child’s therapist/doctor at the children’s service first talk about a transition to adult services?
- Has this been addressed yet?
- How did you feel about this?
- What was discussed prior to the transfer? What made you continue medication/treatment for your child’s ADHD?
- If transition has not yet been addressed, when do you think would be a good time for your child’s doctor to first talk about transition?
- Have you thought about discussing transition and needs into adulthood with your clinician?
- What kind of information have you had from your clinician (other sources) about ADHD and growing up (how it may change, needs, treatment)?
- Do you know how ADHD will progress during adolescence and into adulthood? Has your clinician talked to you about this?
- When did you first have contact with professionals from the adult service?
- If you have not yet had contact, when do you think would be a good time to first have contact?
- How were you, as a parent, involved in this process?
- How, as a parent would you like to be involved in this process?
- How well prepared did you feel for your child’s transfer?
- What would make you feel well prepared for your child’s transfer?
- Is there anything that could have helped you and your child prepare for leaving CAMHS/Paediatrics?
- Is there anything that would help you and your child prepare for leaving CAMHS?

1. **The transition process**

- Is it clear who you can contact for help throughout the process?
- If so, who is this, and how did you find out about them?
- If not, who would you ask to find out?
- Did you have a named contact or transition worker at CAMHS? At the service for adults?
- If still in CAMHS, do you have a named contact in CAMHS, how do you get in touch with them?
- Were there any gaps in your care when you moved from CAMHS to the service for adults, or times when you could not get the help you needed?
- Do you think there may be any gaps in your child’s care when they move from CAMHS to adult services?
- What would do you both do if you could not get the help you needed?
- What did you think about how CAMHS and the service for adults worked together?
- Or if not yet transitioning, how do you think the CAMHS and adult services are likely to work together?
- How would you like them to work together?

1. **Improving transition**

In your opinion, how could transition be improved for young people moving from CAMHS to services for adults?

- What might the best designed service look like?
- What would your child in particular most benefit from during transition?
