# Supplementary material for: In transition with ADHD: the role of information, in facilitating or impeding young people’s transition into adult services
Source: BMC Psychiatry. 2019 Dec 17;19:404. doi: 10.1186/s12888-019-2284-3 (PMC6918680; doi:10.1186/s12888-019-2284-3)
Supplement: Supplementary file 2 — Additional file 2. Recommendations for clinical practice, with supporting quotes. [file 12888_2019_2284_MOESM2_ESM.docx]

# Recommendations for clinical practice, with supporting quotes

## Equip General Practitioners (GPs) so that they understand ADHD as a condition and can signpost to services

GPs are often a first point of contact for young people and may be an essential link to help access services. Therefore they need to be aware of ADHD as a condition and be able to access service information.

The information a GP holds can be a crucial factor in parent/carer and young people’s access to services, medication if required and their well-being. A GPs lack of understanding of ADHD as condition and/or inability to access information about appropriate adult services can be a significant barrier to transition and contribute to young people and parent/carers’ isolation and distress.

## Start sharing key information at least a year in advance

Preparation should be timed so that it can be a staged process with a basic overview of ADHD as a condition plus the need to transition into adult services, followed by more detail. Give young people time to get used to the idea and a chance to discuss options before they reach 18. This enables the young person to increase their understanding of ADHD as a condition, reflect on their own needs and make informed choices about continued engagement with services.

“*I think they need 12 months just to get the idea… to explain everything…we need to forward plan and we need to know what’s going to happen before it happens otherwise the anxiety will just kick in*.” F-P

“*I think it would be useful if … they were spoken to by someone who does a transition type package saying, ‘You are 16 now, there’s lots of options that you can do*.’ *… to have someone who knows about ADHD to say, ‘These are your options’*.” F-0-(P)

Young people with ADHD often said that they did not like sudden changes and wanted time to plan and think about their own futures.

“*I’d rather have time to plan…I don’t like sudden changes.*” F-0

“*I'd rather it sooner than later because then I know what to expect in the future…because it's my future and I don't really know what's going to happen*.” M-0

Preparation needs to be flexible enough to take into account the young person’s developing maturity and how this is interacting with their ADHD.

*“Within that year you'll either see a maturity where they're not growing out of it but learning to live with it shall we say? Or it will be panic stations…let's get them booked in ready and have it all sorted.” M-0-(P)*

## Stage 1. Provide an initial overview of ADHD as a condition and of the transition process

Let the young person know they may or may not continue to be affected by ADHD into adulthood. Let them know transition is a possibility with an overview of how and when it might happen.

“*I'd definitely like to know when and what happens* [at transition]” and when children’s services end.” M-0

“*Could I carry on, if I needed it or something? Would I have to pay for it*?” M-0

Young adults who did not transition recommend that the choice to stop treatment is discussed early, in a way that involves the young person. The clinicians from children’s services should explain that ADHD may cause the young person difficulties into adult life and provide information that they might need to make informed choices about treatment and transition. They recommended a sensible discussion about impact of stopping. They also emphasise the importance of providing young people with the information necessary for re-entry into services if they do lose contact. If someone leaves, they need to know how to access help later.

## Stage 2. Provide nuanced information about the young person’s ADHD needs, combined with detailed information about the transition process

Information about ADHD as a condition is a necessary part of preparation for transition – this might not have been discussed for some years if the young person was diagnosed in childhood. Understanding the way that their ADHD needs may continue, change and develop, the potential impact of study or work environments, and possible use of strategies to deal with this is an important part of empowering young people to manage their own condition and will affect their decisions about continuing into adult services. Most young people and carers would like this information from expert clinicians.

*“I want him to have some help to understand how he can sort himself out and how he can control it himself. There’s only so much I can tell him or he can find out himself*.” F-P

“*He would like a relationship with someone who he can ask these questions of*.” F-P “*Consistency with a knowledgeable, qualified person that can actually help him move into adulthood*.” F-P

Providing detailed information about the transition process, laid out in clear steps may diminish or even avoid uncertainty that can causes high levels of distress in young people. Clear information allows parents/carers to better support young people in navigating organisational information in a way that will allow them to get to their appointments.

Through being provided with detailed information, young people are given an opportunity to reflect and ask questions.

“*I’d have to know what they’d even do to be able to ask questions*.” F-0

## Take ADHD into account when providing information and include parent/carers

People with ADHD struggle with the regulation of attention and organisation. This means they can find it difficult to focus on specific details and may be overwhelmed by too much information. Therefore, information needs to be communicated in simple, clear formats and via several methods. Give the young person time to process new data and avoid discussing lots of things at once.

“*They should take into account the nature of the condition,* [for transition planning]…*I think they understand that you might be slow in your response or that you might not want to sit down all of the time. So they take that into account but they don't when they're giving you loads of information all at once without really repeating it*.” F-P

“*Information leaflets, stuff like that to help you with it and to understand what’s happening or what will happen so it just makes it a lot smoother for you*.” M-1

“*A video would be ideal. Clips would be ideal. Or a picture book, or even just re-enacting. Literally seeing it and re-enacting it out. It needs to be hands-on with people with ADHD.*" M-X

Include and facilitate the parent/carer in their role as information interpreter for the young person. Young people need the option for parent/carer support to continue after leaving child services, given the effect of ADHD on many young people’s ability to organise themselves. The parent/carer is likely to continue to play a crucial role in navigating practical/administrative information to enable the young person to access treatment, as well as supporting them in developing understanding of ADHD as condition and how it may continue to affect them.

## Provide information about being in adult services

Having information about what to expect in the adult service, where it is, who they will be meeting and how treatment may differ from child services is very important to young people. Knowing this in advance helps them manage the process of change and to feel they are ready to engage with the adult service when the time comes.

- Let them know about access into adult services: whether they will qualify for treatment. That there is a service. If there is no service, or they may not meet referral criteria, that is likely to cause distress. However, knowing in advance is better than finding out when they have already left child services.

“*My consultant told me that I probably wouldn’t qualify for the adult psychiatry*…a*t the moment I’m kind of like okay, but…when it was first mentioned I was completely distraught*.” F-1

- Explain differences between child and adult services**:** not knowing this can cause anxiety, while understanding the differences and similarities can help the young person think about how they will be able to manage their condition as an adult, what help will be available and be ready to engage in adult services in a suitable way.

“*I’d like to know how different things would be and how they do things compared to the way that the children services do things.*” F-0

- Share information about the new clinician: if possible provide joint meetings, most young people ask for this, to help them to get to know their new clinician and reduce anxiety around the unknown and meeting new people. If this is not possible, provide as much information about the person as you can.

*“You are integrating into it* [adult service] *it might make things easier rather than it just being a sudden sort of change from this person to this person, if you just slowly met up with them and built a relationship up with them a bit.*” F-0

“*Yeah. I'm not really great around new people.*” M-0

- Share information about the physical location: for young people knowing where they will be going to as an adult, perhaps visiting this place in advance, is a way of managing the process of change.

*“So then you know where you are going and you feel comfortable and you feel acquainted already.”* M-0

1. Share information between child and adult services

Young people with ADHD can struggle with information processing and communication. Making sure the adult clinician and service is already briefed about the young person, which reduces the burden on them to repeat their story.

“*Someone to know a bit more about my past in detail than what I have just said, it would be much better.*” M-0

“*I’d prefer it if a doctor just spoke to the other doctor and actually told her all about it [*me*] and what…the other doctor needs to do…how to handle* [my ADHD].” M-0

## Provide a point of contact

A point of contact when between services is very important both practically and emotionally. Ideally this would be a named person who can update the young person on where they are in the transition process, answer administrative questions and signpost to other services in an emergency. However, even an informed administrator or a drop in centre where they can go to ask questions would make a big difference. This could provide a sense of still being ‘in’ services, rather than left alone. This would help to reduce the stress associated with waiting for adult services which can be very difficult. Hearing nothing at all leads to frustration.

“*Just knowing someone is there if anything was to happen, touch wood it doesn't but if something was to happen I could actually speak to someone.*” M-0

“*Nothing complicated, just if you were to find websites that would be in your face that ‘hey this is the number that you can call anytime’ that would make it easier*.” M-0

*[Note: F=female, M=male. 0=pre-transition; 1=at-transition; X=no-transition (re-entered as adult). P=parent. (P) = Parent commenting within young person’s interview.]*
